# *Ixeris dentata* Extract Increases Salivary Secretion through the Regulation of Endoplasmic Reticulum Stress in a Diabetes-Induced Xerostomia Rat Model

**DOI:** 10.3390/ijms19041059

**Published:** 2018-04-02

**Authors:** Kashi Raj Bhattarai, Hwa-Young Lee, Seung-Hyun Kim, Hyung-Ryong Kim, Han-Jung Chae

**Affiliations:** 1 Department of Pharmacology and Institute of New Drug Development, School of Medicine, Chonbuk National University, Jeonju 54896, Korea; meekasik@jbnu.ac.kr (K.R.B.); youngat84@jbnu.ac.kr (H.-Y.L.); 2 College of Pharmacy, Yonsei Institute of Pharmaceutical Science, Yonsei University, Incheon 406-840, Korea; kimsh11@yonsei.ac.kr; 3 Graduate School, Daegu Gyeongbuk Institute of Science and Technology, Daegu 42988, Korea

**Keywords:** *Ixeris dentata*, diabetes-associated xerostomia, saliva secretion, amylase, aquaporin 5, endoplasmic reticulum stress

## Abstract

This study aimed to investigate the molecular mechanism of diabetes mellitus (DM)-induced dry mouth and an application of natural products from *Ixeris dentata* (IXD), a recently suggested regulator of amylase secretion in salivary cells. Vehicle-treated or diabetic rats were orally treated with either water or an IXD extract for 10 days to observe the effect on salivary flow. We found that the IXD extract increased aquaporin 5 (AQP5) and alpha-amylase protein expression in the submandibular gland along with salivary flow rate. Similarly, the IXD extract and its purified compound increased amylase secretion in high glucose-exposed human salivary gland cells. Furthermore, increased endoplasmic reticulum stress response in the submandibular gland of diabetic rats was inhibited by treatment with the IXD extract, suggesting that IXD extract treatment improves the ER environment by increasing the protein folding capacity. Thus, pharmacological treatment with the IXD extract is suggested to relieve DM-induced dry mouth symptoms.

## 1. Introduction

Xerostomia, also referred to as dry mouth, is one of the main consequences of diabetes mellitus and is induced by salivary gland dysfunction [1]. The destruction of salivary gland tissues leads to the loss of salivary electrolytes and buffers, causing severe oral dryness [2]. *Ixeris dentata* (IXD, Korean name Sseumbagwi) is an herbal medicinal plant used to treat various diseases including diabetes, hepatitis, inflammation, and tumours [3,4,5]. However, its effect on diabetic complications has not yet been studied. Aberrant calcium signalling in the salivary glands leads to dry mouth and other oral complications. Reduced (Ca^2+^)_ER_ in salivary glands leads to improper post-translational processing and folding of endoplasmic reticulum (ER) proteins, consequently decreasing salivary amylase activity [6]. The diminished Sarco/endoplasmic-reticulum Ca^2+^-ATPase (SERCA) was observed in the submandibular gland of STZ-induced diabetic rats which eventually reduced (Ca^2+^)_ER_ [7]. This decreased (Ca^2+^)_ER_ led to the improper protein folding in the salivary gland and exhibited xerostomia including low salivary amylase activity [6]. In addition, hyperglycemia induces perturbation in the intracellular Ca^2+^ (Ca^2+^)i signalling [6] and impairs salivary gland functions and fluid secretion [7,8]. The salivary flow rate and salivary amylase activity have been observed to decrease significantly in diabetes [4,9,10]. mRNA and protein expression of amylase have also been found to be reduced significantly in the parotid glands of diabetic rats [11]. Aquaporin 5 (AQP5) is a water channel protein that plays an important role in water transport during the process of saliva formation and other exocrine secretions [12]. AQP5 expression is impaired in diabetes, resulting in diminished saliva volume. Wang et al. found increased AQP5 mRNA amounts and decreased protein expression in the parotid glands of diabetic rats, suggesting that post-transcriptional dysregulation can lead to a decline in AQP5 proteins [13]. If the translocation of AQP5 to the salivary gland is decreased, the downregulation of protein expression leads to dry mouth symptoms.

The ER is an organelle with roles in the biosynthesis of secretory proteins, such as amylase, and in protein folding. Alpha-amylase requires a proper folding environment in the ER for its maturation and secretion. It exits through a particular subdomain of the ER [14]. Any perturbation between its folding capacity and the physiological load of secretory protein translocated into the ER can lead to ER stress [15]. ER stress is a process associated with impaired physiological function where unfolded proteins accumulate and induce the unfolded protein response [16]. Under ER stress conditions, GRP78 engages with unfolded proteins and is released from ER stress sensors like IRE1α, PERK, and ATF6α. Dissociation of GRP78 leads to dimerization and activation of IRE1α [5]. Activation of IRE1 cleaves XBP-1 into its active (spliced) form. The active form of XBP-1, a transcription factor, boosts the transcription of endoplasmic reticulum-associated protein degradation (ERAD) target genes such as endoplasmic reticulum degradation-enhancing alpha mannosidase like protein (EDEM) [17]. PERK, activated by trans-autophosphorylation and dimerization, phosphorylates the α-subunit of eIF2α and hinders global protein synthesis [18]. Furthermore, it induces the transcription factor ATF4 which later induces another important target gene, CHOP [19]. Dry mouth and inflammation of salivary glands is associated with increased salivary gland expression of GADD153/CHOP, suggesting an important role for ER stress response in the pathogenesis of Sjogren’s syndrome [20]. It has been observed that increased GADD153 in the kidneys and blood cells of db/db mice upregulates the ER stress response [21]. ER stress-induced autophagy and apoptosis have been observed in thapsigargin-treated human salivary gland cells [22]. Upon ATF6α activation, it translocates to the Golgi apparatus and leads to cleavage from the membrane by S1P and S2P (site-1 protease and site-2 protease) [23]. High expression of inflammatory cytokines leads to the activation of ATF6α in the salivary glands of SS patients [24]. Some statements from previous studies reveal that although the activation of ATF6α is increased, there is a possibility for the reduction in the activity of IRE1α, suggesting a state of chronic ER stress in the salivary glands of SS patients [24,25]. Depending on cellular fate, persistent ER stress can lead to IRE1-induced JNK activation [26] or attenuate IRE1 signalling by promoting de-phosphorylation of IRE1α and by decreasing endoribonuclease activity [27,28]. The in vitro application of the IXD extract has been identified as an effective treatment to reduce hepatic dyslipidaemia through the regulation of ER stress [29]. Our previous study has suggested that the IXD extract increases the folding and synthesis of amylase by regulating ER stress in human salivary gland (HSG) cells on exposure to high glucose levels [4,5].

The exact molecular mechanism for hyposalivation and diabetes-induced xerostomia is still unclear. This study investigated the underlying molecular mechanism and a possible treatment using the IXD extract in diabetic rats.

## 2. Results

### 2.1. Efficacy of Active Compound from IXD Extracts and Effect on Amylase Expression

**Figure 1:**
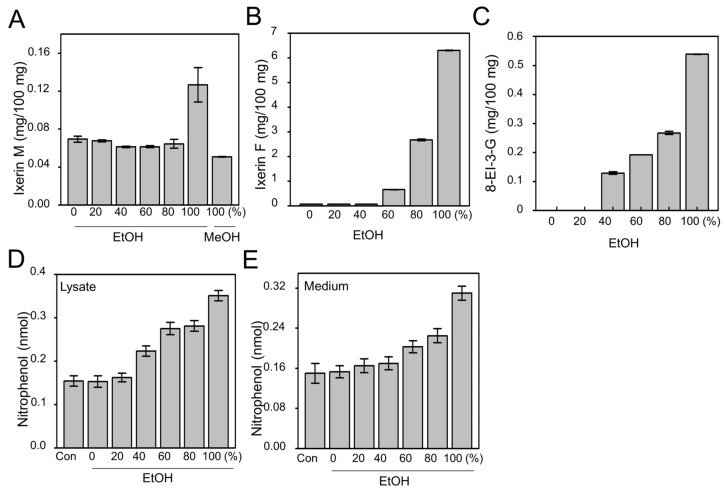
The effect of the different grades of ethanol on extraction of IXD. Purified compounds, (**A**) Ixerin M; (**B**) Ixerin F; (**C**) epiisolipidiol-3-β-d-glucopyranoside (8-EI-3-G) in the IXD extract found by extraction with different grades of ethanol and 100% methanol; (**D**,**E**) effect of different grades of ethanol on extraction of IXD and measurement of amylase secretion in HSG cells exposed to high glucose concentrations; (**D**) amylase secretion measurement using cell lysates; (**E**) amylase secretion measurement using cell cultured medium.

IXD was extracted in different grades of ethanol (0%, 20%, 40%, 60%, 80%, and 100%) and 100% methanol. The 100% ethanol-extracted IXD efficiently yielded high amounts of Ixerin M, Ixerin F and 8-epiisolipidiol-3-β-d-glucopyranoside (8-EI-3-G), the major components of IXD, as compared to low grades of ethanol (Figure 1A–C). A chromatogram of Ixerin M, Ixerin F and 8-EI-3-G is shown in Figure S1B–D. 100% ethanol-extracted IXD had increased amylase secretion by almost two-fold in both cell lysates and medium as compared with IXD untreated cells. IXD extracted with 100% EtOH had higher amylase than when extracted with low grades of ethanol (Figure 1D,E), suggesting that 100% ethanol extract of IXD had significant influence on amylase extraction. Based upon these data, we chose 100% ethanol IXD extract for the current study.

### 2.2. IXD Extracts Increase Amylase Synthesis and Secretion in High Concentration Glucose Treated Human Salivary Gland Cells

**Figure 2:**
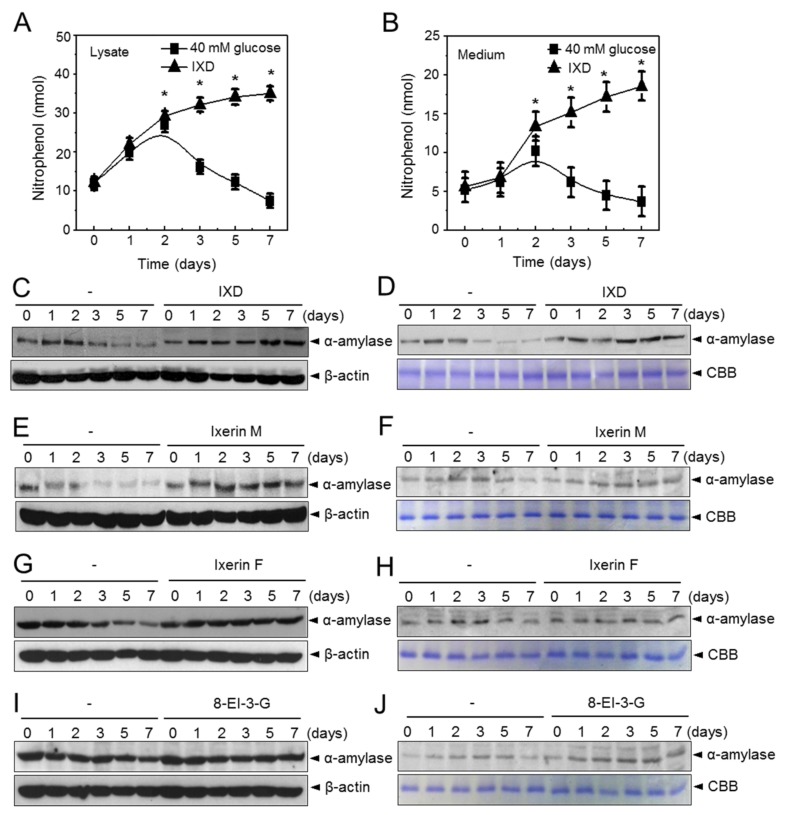
The IXD extract increases amylase synthesis and secretion in human salivary gland cells exposed to high glucose levels. (**A**,**B**) Amylase activity was measured in cell lysates (**A**) and cultured medium (**B**) of HSG cells exposed to high glucose concentrations with or without the IXD extract for 1, 2, 3, 5, and 7 days. Values are represented as mean ± SEM (*n* = 3, *p* < 0.05). * indicates significant differences with the IXD-untreated cell condition; (**C**,**D**) amylase expression in cell lines exposed to high glucose concentrations treated with or without the IXD extract for the indicated time period; (**C**) amylase protein expression was analysed using cell lysates; (**D**) amylase protein expression was analysed using cell cultured medium; (**E**,**F**) HSG cells were exposed to high glucose concentrations (40 mM) for 1, 2, 3, 5, and 7 days in the presence or absence of Ixerin M, a purified IXD component, and amylase expression was measured in cell lysates (**E**) and cell medium (**F**); (**G**,**H**) amylase protein expression was analysed in cell lysates or cultured medium in the presence or absence of Ixerin F and (**I**,**J**) epiisolipidiol-3-β-d-glucopyranoside (8-EI-3-G).

The IXD extract and control were used to treat high glucose-exposed human salivary gland (HSG) cells for 1, 2, 3, 5, and 7 days. Chronic exposure of HSG cells to high glucose led to restriction in amylase secretion. Amylase secretion in both cell lysates and cultured medium was gradually decreased after the second day of high glucose treatment. When IXD was applied in the same cellular condition, we observed a significant (*p* < 0.05) increase of amylase activity in cell lysates (Figure 2A) as well as in cultured medium (Figure 2B). Furthermore, we checked protein expression by Western blot and found that α-amylase protein expression was highly increased in both cell lysates (Figure 2C) and medium (Figure 2D) in IXD-treated high glucose-exposed HSG cells. Furthermore, to check amylase expression for IXD compounds such as Ixerin M, Ixerin F and 8-EI-3-G, we cultured HSG cells containing a high glucose concentration (40 mM) and treated with compounds for 1, 2, 3, 5, and 7 days. Amylase expression was increased to a greater extent in both the cell lysates (Figure 2E,G,I) and medium (Figure 2F,H,J) of IXD compounds treated HSG cells than those exposed with only a high concentration of glucose. These data suggest that the diminished α-amylase secretion by hyperglycaemia can be restored with the help of IXD extract treatment.

### 2.3. The Effects of the IXD Extract on Salivary Parameters

**Figure 3:**
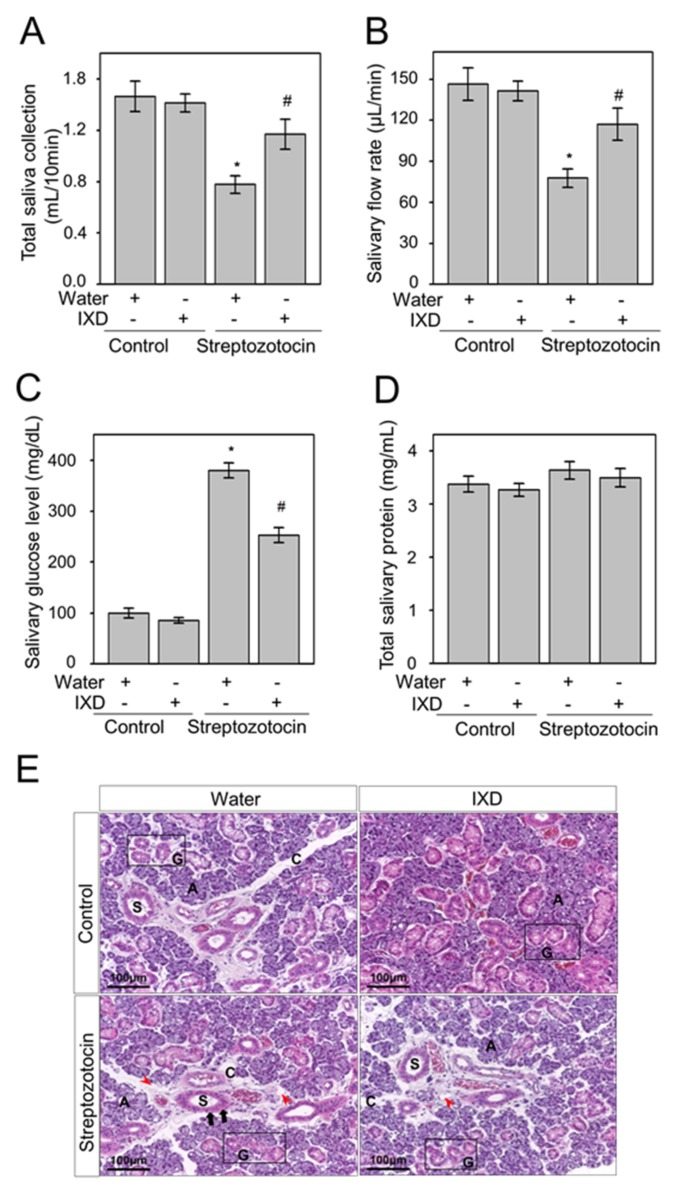
The effects of the IXD extract on salivary parameters. (**A**) Total salivary volume (mL) collected at 10 min after pilocarpine injection; (**B**) the total salivary flow rate was measured in μL/min; (**C**) measurement of glucose concentration in saliva; (**D**) measurement of total protein concentration in saliva (mg/mL). Values are represented as mean ± SEM (*n* = 10 per group, *p* < 0.05). * indicates significant differences compared to vehicle-treated control rats and # indicates significant differences compared to STZ-induced diabetic rats; (**E**) haematoxylin and eosin staining showing the morphological appearance of the submandibular glands of control and diabetic rats. Red arrowheads show variable degrees of vacuolization of the connective tissue stroma. Black arrows show slightly dilated and pyknotic nuclei in striated ducts. Characters showing in pictures such as A, C, S and G denotes for acinar cells, connective tissue stroma, striated ducts and granular convoluted tubules respectively. Magnification: 20×; Scale bar: 100 μm.

It has been reported that hyperglycaemia is associated with decreased salivary secretion [30] and leads to the complication of dry mouth [31]. Consistent with these previous findings, we observed significant reduction (*p* < 0.05) in salivary secretion in STZ-induced diabetic rats. There were no significant changes in saliva secretion in water or IXD-treated control rats. Treatment with the IXD extract for 10 days significantly (*p* < 0.05) increased salivary secretion and salivary flow rate in diabetic rats (Figure 3A,B). These results suggest that the IXD extract induces saliva secretion and prevents hyposalivation-induced dry mouth condition. Similarly, submandibular gland weight was significantly reduced in STZ-diabetic rats (*p* < 0.05), however, IXD treatment showed an improved pattern in diabetic rats (Figure S2D). We further analysed glucose concentrations in saliva and found that the increased glucose level in diabetic saliva was reduced significantly (*p* < 0.05) in IXD-treated diabetic rats. There were no visible differences in IXD-treated or untreated control rats (Figure 3C). We found no significant differences in total protein concentration in saliva of IXD-treated diabetic or control rats (Figure 3D). Furthermore, we performed H and E staining to observe the morphology of submandibular gland (Figure 3E). The acinar cells of submandibular glands were not disturbed in vehicle or IXD-treated control rats. We observed variable degrees of cytoplasmic vacuolization (majority enlarged) in acinar cells and vacuolization of the connective tissue stroma in diabetic rats. Similarly, slightly dilated and pyknotic nuclei were observed in striated ducts. Atrophied and irregular granular convoluted tubules were observed in diabetic rats, which might have led to secretory dysfunction. Cell morphology was partly regenerated by IXD extract treatment and eventually recovered its normal function (Figure 3E). These data suggest that the regular oral administration of IXD extract could improve the morphology of damaged glandular cells and improve the function of submandibular gland.

### 2.4. The Effects of the IXD Extract on Salivary Amylase Expression

**Figure 4:**
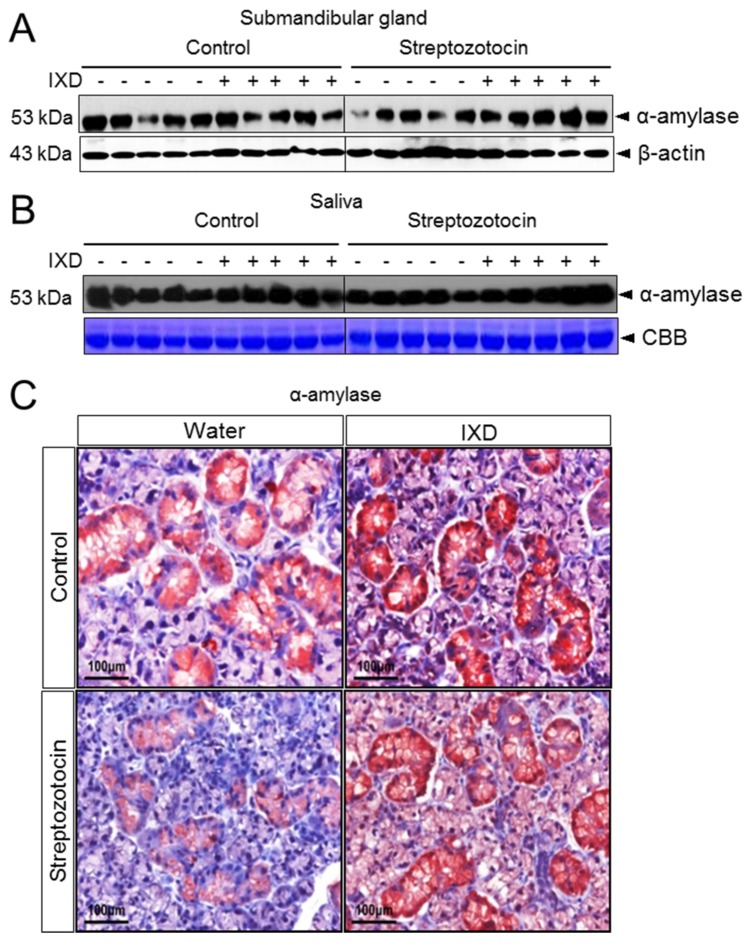
Expression of α-amylase in the submandibular gland and saliva. (**A**) Amylase expression in submandibular gland tissue lysates; (**B**) amylase expression in saliva; (**C**) paraffin-embedded submandibular gland tissues were sectioned at 5 μm thickness, and immunostaining was performed using an amylase antibody. Brownish red colours indicate immunolocalization of α-amylase in submandibular gland cells. Magnification: 40×; Scale bar: 100 μm.

There were no differences in amylase expression of IXD-treated and untreated control rats but it was decreased in the submandibular gland tissue of diabetic rats (Figure 4A). Amylase expression in saliva was also reduced in diabetic rats when compared with the control group (Figure 4B). However, treatment with the IXD extract increased amylase expression in both salivary gland tissue homogenates (Figure 4A) and in saliva (Figure 4B). Various conflicting results have been found for the expression of α-amylase in the submandibular gland. One previous study stated that α-amylase is predominantly localized in epithelial cells of the granular convoluted tubule cells. Another study suggested that immunoreactive expression of α-amylase was detected in the secretory granules of submandibular gland acinar cells but not in GCT cells [32]. During our study, we observed that α-amylase was highly expressed in the acinar cells and somewhat expressed in duct cells. Control rats treated with either vehicle or IXD showed abundant expression of α-amylase in the submandibular gland (Figure 4C). The submandibular gland of diabetic rats showed lower expression of α-amylase; conversely, expression was increased drastically by treatment with the IXD extract (Figure 4C). Immunofluorescence data suggested that the localization of α-amylase and co-localization with AQP5 was reduced in acinar cells of the diabetic submandibular gland; however, it was restored with the treatment of the IXD extract (Figure S4). These results suggest that the IXD extract helps to produce salivary fluid and α-amylase from acinar cells, which secrete through a ductal network towards the oral cavity, and to subsequently aid recovery of hyposecretion from the salivary gland.

### 2.5. The IXD Extract Increases Fluid Secretion through the Activation of AQP5 and NHE1 in the Submandibular Gland

**Figure 5:**
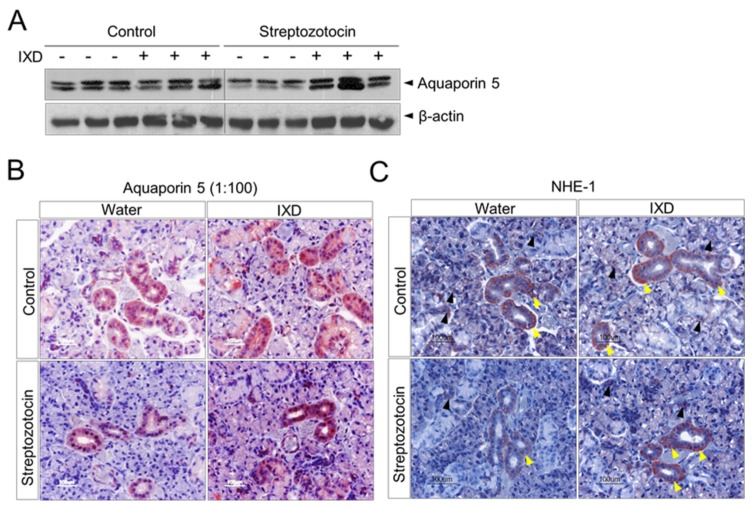
Expression of AQP5 and NHE1 in the submandibular gland. (**A**) Western blot analysis showing AQP5 protein expression in submandibular gland tissue lysates. The blot identified two bands of AQP5 (27 kDa and 31 kDa) revealing non-glycosylated and glycosylated forms of the AQP5 protein respectively; (**B**) the expression of AQP5 (1:100) was performed on submandibular gland tissue sections of either water or IXD extract-treated control or diabetic rats. Brownish red colour indicates the positive expression of AQP5. Magnification: 40×; Scale bar: 100 μm; (**C**) immunohistochemical detection of NHE1 in submandibular gland tissue from normal and diabetic rats treated with water or the IXD extract. Yellow arrow heads show the expression of NHE1 in duct cells, whereas black arrow heads indicate acinar expression. Magnification: 40×; Scale bar: 100 μm.

We performed Western blot analysis to check AQP5 protein expression in submandibular gland homogenates of normal or diabetic rats. We found higher expression of AQP5 in water- or IXD-treated normal rats. AQP5 protein expression was downregulated in STZ-induced diabetic rats, suggesting hyposecretion of saliva. However, the expression of AQP5 was enhanced after IXD treatment in diabetic rats, indicating a positive role for the IXD extract on water channel activation (Figure 5A). The localization of AQP5 has various contradictory results as reviewed by [33]. To ascertain the expression of AQP5 in submandibular gland, we performed immunostaining using two different concentrations of antibodies (1:100 and 1:500). A high concentration of antibody showed strong immunoreactivity in both the apical and lateral membrane areas of serous and mucous acinar cells and in ductal cells (Figure 5B), whereas a low concentration of antibody stained mainly in the acinar cells with weak expression observed in ductal cells (Figure S3). The expression pattern did not differ between IXD-treated or untreated healthy tissues. Furthermore, the diabetic rats showed faint expression in both the acinar and ductal cells of the submandibular gland when compared with control rats. Treatment of diabetic rats with the IXD extract increased AQP5 expression with continuous and uniform distribution (Figure 5B and Figure S3). Similarly, co-localization of AQP5 with α-amylase was attenuated in submandibular gland acinar cells of diabetic rats and was restored in IXD-treated diabetic rats (Figure S4).

NHE1 is thought to act as a regulator of intracellular pH in acinar cells and also participates in saliva secretion. Reduced saliva volume has been observed in NHE1-deficient mice [34,35]. In this study, we performed immunostaining using NHE1 antibody to investigate the effect of the IXD extract on NHE1 expression. We observed that well expressed NHE1 predominantly localized to the submandibular gland duct cells and in acinar cells of control rats. Diabetic rats showed weak expression of NHE1 in the ductal and acinar cells when compared with control rats. Surprisingly, the expression of NHE1 was subsequently increased in ductal and acinar cells of the diabetic submandibular gland in response to IXD treatment (Figure 5C), suggesting a role of IXD extracts in NHE1-induced salivation.

### 2.6. The IXD Extract Regulates ER Stress-Induced Hyposalivation in Diabetic Rats

**Figure 6:**
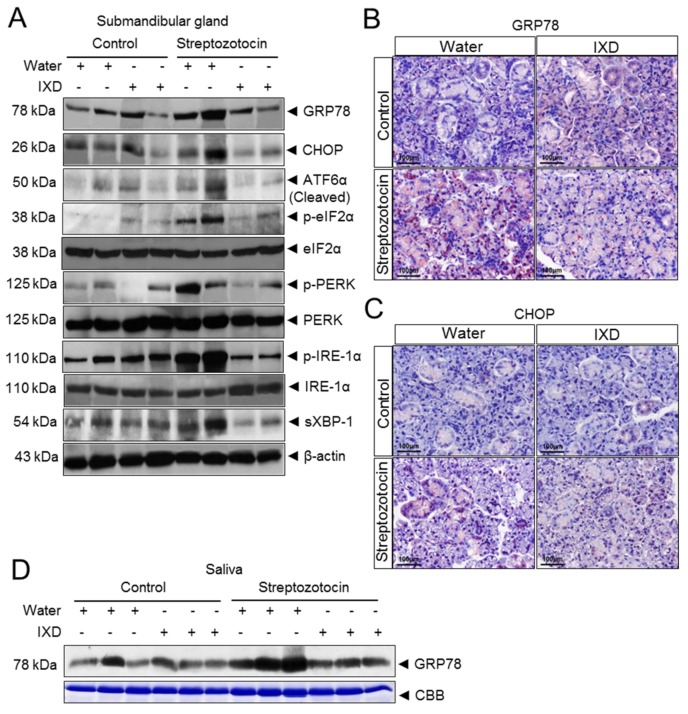
The IXD extract regulates the ER stress response in submandibular gland of diabetic rats. (**A**) Western blot analysis was performed to demonstrate the ER stress response using GRP78, CHOP, ATF6α, p-eIF2α, total eIF2α, p-PERK, total PERK, p-IRE1α, total IRE1α, and sXBP-1 antibodies. An equal concentration of protein (30 μg) was loaded in each lane of the gels. Beta-actin was used as a loading control. The molecular weight of each protein is indicated in kDa; (**B**) immunostaining of GRP78; (**C**) immunostaining of CHOP in the submandibular gland. Magnification: 40×; Scale bar: 100 μm; (**D**) expression of secreted GRP78 in saliva in the indicated groups. Coomassie Brilliant Blue (CBB) staining was performed as a control for equal protein loading.

In another set of experiments, immunoblot assays were performed using submandibular gland tissue lysate to measure the expression of several ER stress markers such as glucose-regulated protein 78 (GRP78), CCAAT enhancer binding protein homologous protein (CHOP), activating transcription factor 6 alpha (ATF6α), phosphorylated eukaryotic translational initial factor 2 alpha (p-eIF2α), phosphorylated protein kinase R like endoplasmic reticulum kinase (p-PERK), phosphorylated inositol-requiring enzyme 1 alpha (p-IRE1α), and spliced X box binding protein 1 (sXBP1). We observed no differences in protein expression of ER stress markers in vehicle and IXD-treated control rats. The expression of ER stress response proteins GRP78, CHOP, ATF6α, p-PERK, p-eIF2α, p-IRE1α, and sXBP1 was upregulated in STZ-induced diabetic rats (Figure 6A). To investigate the underlying mechanism of the IXD extract on ER stress inhibition, we observed a reduction of these ER stress proteins, suggesting that the IXD extract can reduce ER stress response in diabetic submandibular gland (Figure 6A). Moreover, we performed immunostaining to observe the expression of GRP78 and CHOP in submandibular glands and found that GRP78 was strongly expressed in the diabetic submandibular gland acinar cells. IXD treatment reduced the expression of GRP78 in diabetic submandibular gland acinar cells, strongly suggesting that IXD treatment regulates the ER stress observed in diabetic rats (Figure 6B). The high expression of CHOP observed in diabetic submandibular gland was also downregulated after the treatment with the IXD extract (Figure 6C). Furthermore, the most fascinating finding we observed was the overexpression of GRP78 in the saliva of diabetic rats when compared with its counterparts. The IXD extract lowered the secretion of GRP78 in the saliva of diabetic rats (Figure 6D).

### 2.7. The IXD Extract Improves Protein Folding and Reduces Oxidative Stress Induced by Diabetes

**Figure 7:**
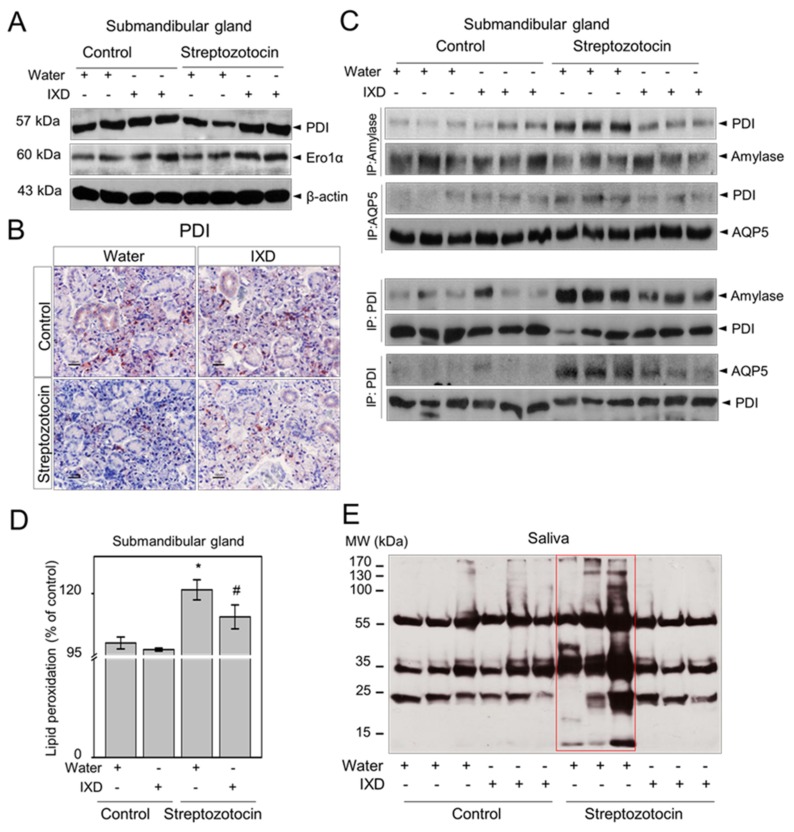
The IXD extract regulates ER folding environment. (**A**) Western blot analysis showing the expression of PDI and ERO1α in submandibular gland lysates; (**B**) immunostaining of PDI in the submandibular gland. Magnification 40×; Scale bar: 100 μm; (**C**) submandibular gland lysates were immunoprecipitated with anti-amylase or AQP5 antibody and immunoblotted with the anti-PDI (upper), anti-amylase or anti-AQP5 antibody (lower); (**D**) TBARS assay was performed to measure lipid peroxidation level in vehicle or IXD-treated submandibular gland homogenates. Values are expressed as percentage of control, (*n* = 10 per group, *p* < 0.05). * indicates significant differences compared to vehicle-treated control rats and # indicates significant differences compared to STZ-induced diabetic control rats; E: Detection of salivary carbonyls using Western blotting. The molecular weight (MW) is indicated in kDa. Red rectangular box shows the high protein carbonylation pattern in the saliva of STZ-induced diabetic rats.

Protein disulphide isomerase (PDI) acts as a chaperone and folding catalyst to repair protein disulphide bond formation and is involved in protein folding activity in the ER [36]. Endoplasmic reticulum oxidoreductin 1 alpha (ERO1α) helps to maintain redox homeostasis by regulating redox potential in the ER [37]. In the present study, we observed less expression of PDI and ERO1α in diabetic rats when compared with healthy rats (Figure 7A), suggesting that hyperglycaemia could impair oxidative protein folding in the ER. However, the reduced protein expression of PDI and ERO1α in the submandibular gland of diabetic rats was upregulated with IXD extract treatment (Figure 7A). Immunohistochemistry data reveals that acinar cell-localized PDI expression was relatively low in the submandibular gland of diabetic rats. The expression of PDI in submandibular gland duct cells was also very weak in diabetic rats when compared with control rats (Figure 7B, bottom left). Importantly, diabetic rats treated with the IXD extract increased the expression of PDI in both acinar and duct cells of the submandibular gland (Figure 7B, bottom right). Interaction between PDI and amylase or PDI and AQP5 was examined by using immunoprecipitation techniques. We observed that PDI was tightly bound to amylase in the submandibular gland of diabetic rats, whereas PDI was clearly dissociated from amylase in the submandibular gland of IXD-treated diabetic rats (Figure 7C, above). Similarly, the association between PDI and AQP5 was increased in the diabetic submandibular gland as compared with the IXD-treated diabetic group (Figure 7C, above). Furthermore, the interaction between amylase or AQP5 with PDI was stable and remained complexed in the diabetic condition and was clearly dissociated in IXD-treated group (Figure 7C, below). These findings suggest that PDI stably binds with amylase and AQP5, or amylase and AQP5 fail to dissociate with PDI. This increases misfolded proteins in the submandibular gland of diabetic rats, whereas IXD assists to increase proper folding.

Furthermore, we analysed oxidative stress markers including lipid peroxidation and salivary protein carbonylation. The lipid peroxidation level was greatly increased (*p* < 0.05) in the submandibular gland of diabetic rats compared with their respective controls. Surprisingly, the administration of the IXD extract significantly reduced (*p* < 0.05) lipid peroxidation levels in submandibular gland tissue samples with respect to the diabetic groups (Figure 7D). In addition, the Western blot results presented in Figure 7E clearly indicate a high level of carbonylation in the saliva of diabetic rats as compared with controls. Diabetic rats treated with the IXD extract had lower levels of protein carbonyls in the saliva (Figure 7E).

## 3. Discussion

Attenuation of salivary flow leads to pathological thirst [38] and results in more concentrated saliva with increased osmolality and protein concentration [39,40]. A previous study done by Mandel et al. found that individuals with higher amylase had significantly lower post-prandial blood glucose levels following starch ingestion when compared with lower amylase individuals [41]. Hypoglycaemic and hypolipemic effects of IXD have been reported in previous studies [42]. Likewise, the regulatory effect of IXD on hepatic dyslipidaemia through the regulation of ER stress has also been reported [29]. During our study, we observed lower concentrations of saliva and blood glucose in IXD-treated diabetic rats (although not in normal range) as compared with their control counterparts (Figure 3D and Figure S2B respectively). This may be due to the influence of IXD extracts to increase high secretion of amylase, or IXD extracts have anti-diabetic effects improving hyperglycaemia. This difference is not elucidated in this study. Although a systemic effect was observed in IXD-treated diabetic rats, local effects for salivary secretion were the focus in the present study. In contrast, total body weight was also improved when IXD extracts were applied to diabetic rats (Figure S2C). Alpha amylase has been used to measure secretory protein biosynthesis and it was found that total α-amylase activity in tunicamycin-treated cell suspensions was greatly reduced [43]. (Ca^2+^)_ER_ plays a vital role in the regulation of saliva and amylase secretion. Intracellular Ca^2+^ signalling is decreased in the salivary gland of STZ-induced diabetic rats and this decrease impairs the metabolic homeostasis of the ER. This in turn affects the synthesis, folding, and maturation of secretory proteins (e.g., amylase) [4,7].

Chronic high glucose treatment of HSG cells has been reported to induce classical ER stress response and eventually reduce the secretion of amylase [5]. In the present in vivo study, we observed that the decreased expression of AQP5 in submandibular gland led to the reduction of saliva secretion in diabetic rats. Consequently, amylase synthesis and secretion were lowered and caused dehydration. The IXD extract highly increased salivary secretion in the submandibular gland by increasing AQP5 and resulted in high amylase expression (Figure S4) and represents a potential target to improve xerostomia. Acinar-cell-localized AQP5 increases water permeability and has an important role in rapid mobilization of water in salivary glands. Previous studies revealed that the AQP5 expression was also observed in the apical portion of the intercalated duct cells of rat submandibular glands which suggested that AQP5 may have a role in the absorption or secretion of small solutes [44]. Furthermore, we examined the expression of NHE1 since it has an important role in NaCl absorption [34]. In addition, NHE1 has a vital role in intracellular pH regulation and saliva production [35,45]. Increased intracellular calcium is one of the main contributors for the muscarinic-stimulated upregulation of NHE1 activity [35]. Acinar-cell-localized NHE1 contributes to salivary fluid and electrolyte secretion, whereas localization to the duct cells increases NaCl reabsorption (Figure 5C). Mice with NHE1 deficiency decrease the secretion of pilocarpine-stimulated saliva, suggesting an important role of NHE1 in salivary secretion [34]. Moreover, genetic loss of NHE1 increases renal tubular apoptosis and induces renal dysfunction in STZ-induced diabetes, indicating NHE1 activity has a beneficial effect on chronic kidney disease [46].

Decreased expression of AQP5 in the submandibular gland of diabetic animals might be the consequence of oxidative stress in the salivary gland. In addition, ER stress response has also been observed in diabetic submandibular glands indicating that activation of ER stress signalling pathways consequently decreases AQP5 expression and increases salivary gland dysfunction. Reduced AQP5 protein expression and upregulated, phosphorylated JNK have been observed in submandibular glands of radiation-induced sialadenitis rat model, causing several oral complications (e.g., xerostomia) [47]. The ER lumen has an ability for post-translational processing and folding of secreted proteins [48]. When the cellular environment is disturbed, proteins cannot fold properly, and these unfolded proteins accumulate in the ER, leading to an ER stress response in cells [49]. Our previous study revealed that the transient treatment with IXD extract augmented the (Ca^2+^)i in HSG cells, suggesting the role of IXD in salivary secretion [4]. In our current study, we focused on the ER folding environment and found that the ER folding capacitance was decreased in the salivary gland of diabetic rats. Interestingly, IXD extract increased the expression of PDI and ERO-1α, suggesting the role of IXD to improve proper protein folding in the ER. These data reflect that IXD may have an ability to refill the (Ca^2+^)_ER_ storage and participates in ER homeostasis.

ER chaperone BiP (binding immunoglobulin protein, also called GRP78) belongs to the HSP70 multigene family and is generally increased during ER stress [16,50]. Under stress conditions, it can migrate to the cell surface and into the extracellular space. There is much evidence that GRP78 can act as an autoantigen in disease and its expression is increased in whole saliva and the synovium of rheumatoid arthritis patients [51,52]. Thus, BiP antigen acts as a pro-inflammatory factor and triggers immunological responses including inflammation [52]. In the present study, the most intriguing finding observed was the extensive release of GRP78 in the saliva of diabetic rats (Figure 6D), suggesting that extracellular GRP78 likely increases pro-inflammatory cytokines and triggers chronic inflammation. Diabetic rats treated with the IXD extract limited GRP78 secretion in saliva (Figure 6D), suggesting a possible inhibitory action against stress.

In the present study, we applied IXD extracts and observed significant increases in salivary flow rate (Figure 3B) and α-amylase expression (Figure 4) in the submandibular gland and in the saliva of diabetic rats. The IXD extract and its purified compound enhanced oxidative folding-associated protein patterns in high glucose treated HSG cells, such as PDI and ERO-1α, and alleviated ER stress [5]. Consistent with our previous in vitro study, we observed high expression of PDI and ERO1 in IXD-treated diabetic rats (Figure 7A,B), suggesting increased oxidative protein folding capacity in the ER environment. PDI was not clearly dissociated from amylase and AQP5 but was rather tightly bound (Figure 7C, above). In a similar fashion, amylase and AQP5 were also stably bound with PDI and could not dissociate from PDI (Figure 7C, below), leading to misfolded proteins in the diabetic condition. This may increase lipid peroxidation levels in the submandibular gland (Figure 7D) and generate high protein oxidation in the saliva (Figure 7E). GSH depletion and irradiation, generate ROS and lipid peroxidation. This leads to increased oxidative stress and, in turn, to salivary gland dysfunction and dry mouth [1,53,54]. IXD is well known to contain high antioxidant properties and has been found to reduce oxidative stress by retaining glutathione concentrations in human salivary gland cells [5] and in mice brain [55]. In this study, we observed significantly higher levels of lipid peroxidation, a strong marker for oxidative stress in diabetic rat submandibular glands. This was further alleviated by treatment with the IXD extract (Figure 7D). Similarly, salivary carbonyls were observed to be increased in the diabetic group and markedly decreased by IXD treatment (Figure 7E). The IXD extract has been shown to have effective antioxidant potential, to reduce oxidative stress exacerbated by hyperglycaemia (Figure 7D,E), and to maintain sufficient salivary enzyme secretion (e.g., amylase) (Figure 4). The IXD extract and the purified compounds have shown capacity to increase amylase secretion in hyperglycaemic conditions (Figure 2), signifying the importance of antioxidant enzyme defences in the salivary glands of diabetic rats. Higher salivary amylase shows a protective function against oxidative stress in the present study and as stated in a previous article [54].

During our study, oral gavage treatment with the IXD extract to diabetic rats showed an increase in salivary secretion. The expression of α-amylase, AQP5, and NHE1 were highly active in IXD-treated rats. ER stress markers such as GRP78, CHOP, ATF6α, p-PERK, p-eIF2α, p-IRE1α, and XBP-1 in diabetic rats were downregulated with treatment with the IXD extract, suggesting that salivary secretion is influenced by stress occurring in the ER. The IXD extract alleviated the ER stress response and balanced the protein folding environment leading to proper functioning of the submandibular gland. Importantly, the increased expression of PDI and ERO1α and decreased expression of ER stress related proteins improved ER protein folding capacity and led to salivary gland homeostasis. These data suggest that ER stress-associated diabetes-induced hyposalivation can be reversed effectively by treatment with the IXD extract. The response observed during saliva secretion may be connected with increased parasympathetic and sympathetic activity since the main neuronal regulation of saliva secretion is by both parasympathetic and sympathetic nerve endings. The parasympathetic neurons mainly stimulate fluid secretion by releasing acetylcholine and increasing (Ca^2+^), whereas the sympathetic nervous system activates β-adrenergic receptors in acinar and ductal cells and increases cAMP [56]. Linking ER stress and neuronal regulation of salivary secretion would be a subject for future research.

## 4. Materials and Methods

### 4.1. Plant Materials and Preparation

The roots of *Ixeris dentata* were harvested in Dangin, Korea in March 2014. The voucher specimens (ID 2014-01) were identified at the National Institute of Horticultural and Herbal Science, Rural Development Administration, Korea, and deposited at the College of Pharmacy, Yonsei University, Incheon, Korea. The dried roots of *Ixeris dentata* were chopped into smaller pieces and further ground into powder. The powder (1.2 kg) was extracted three times with 3 L of ethanol in an ultrasonic apparatus for 3 h at 35° C. Removal of the solvent in vacuo yielded an ethanol extract (30.5 g, 2.5% dry weight, also summarized in Table S1). The ethanolic extract was suspended in water to the desired concentration before use.

### 4.2. Quantitation of Pure Compound Using HPLC-DAD

An *Ixeris dentata* extract was standardized by using HPLC-DAD. The HPLC system consisted of a Waters Acquity UPLC H-Class System (Waters, Milford, MA, USA) and the output signal was recorded using Empower^TM^ Software (version 2, Waters, Milford, MA, USA). In brief, chromatographic separation was carried out using a linear gradient elution of acetonitrile (A) and 0.1% acetic acid in water (B) (0 min, 5% A; 20 min, 25% A; 20.1 min, 5% A; 30 min, 5% A) on an Inno C18 column (Innopia, Gyeonggi-Do, Korea) (4.6 × 150 mm, 5 μm) at a flow rate of 1.0 mL/min. The wavelength for quantification of the IXD components were set at 254 nm (also summarized in Figure S1A).

### 4.3. Chemicals and Reagents

Streptozotocin (STZ), citric acid, and pilocarpine hydrochloride were obtained from Sigma Chemical Company (St. Louis, MO, USA).

### 4.4. Cell Culture and Treatment

Human salivary gland (HSG) cells were obtained from the American Type Culture Collection (ATCC). Cells were cultured in RPMI 1640 media as described in [5]. HSG cells were treated with 40 mM glucose in the presence or absence of Ixerin M (0.002 mg/mL), Ixerin F (0.002 mg/mL), and 8-EI-3-G (0.002 mg/mL) an active compound found in the IXD extract for 1, 2, 3, 5, or 7 days. Furthermore, HSG cells were treated with 40 mM glucose in the presence or absence of ethanol-extracted IXD (0.02 mg/mL) for 1, 2, 3, 5, and 7 days. Amylase activity and expression were analysed using cell lysates and cell cultured medium.

### 4.5. Animals

Forty Sprague-Dawley (SD) rats (7-week males, 220–250 g) were obtained from Damul Science, Daejeon, South Korea and used in the study. They were divided into 4 groups and acclimatized to laboratory conditions for 1 week at a temperature of 22 ± 2 °C, 55–60% relative humidity, and 12 h light/dark cycle before use in the experiments. A tap water and standard pellet diet was freely accessible throughout the experimental period. All animal care and experiments were carried out in accordance to the institutional guidelines of Chonbuk National University and were approved by the Institutional Animal Care and Use Committee (IACUC) (Approved No.: CBNU 2017-0015).

### 4.6. Experimental Design

The schematic diagram of the in vivo experiment is shown in Figure S2A. In brief, animals were divided into four groups as follows.

Group 1: Control rats treated with vehicle (Control + water);

Group 2: Control rats treated with the IXD extract (Control + IXD);

Group 3: Diabetic rats treated with vehicle (STZ + water);

Group 4: Diabetic rats treated with the IXD extract (STZ + IXD).

After three days of STZ or vehicle injection, rats were screened for diabetes. Next day, vehicle or diabetic rats were treated with either water or the IXD extract (100 mg/kg body weight) via oral gavage for 10 days. Two-hour fasted, anesthetized rats were injected with pilocarpine hydrochloride (0.6 mg/kg, IP) and saliva was collected for 10 min using pre-weighed cotton balls. Rats were then sacrificed and the bilateral submandibular gland was isolated and weighed. One lobe was frozen and stored at −80 °C and another lobe was kept in formalin (Formaldehyde 3.7%, Dana, Seoul, Korea) for histological analysis.

### 4.7. Induction and Assessment of Blood and Saliva Glucose Level

Diabetes was induced in rats by a single intraperitoneal dose of freshly prepared STZ (65 mg/kg, dissolved in 100 mM cold citrate buffer, pH 4.5). Vehicle control rats were injected with citrate buffer only. Three days after STZ injection, rats were assessed for diabetes (hyperglycaemia with blood glucose concentrations higher than 300 mg/dL). Blood glucose was measured in a tail vein using a glucometer (Accu Chek, Roche, Mannheim, Germany). The salivary glucose level was determined using a glucose colorimetric assay kit (Bio Vision, Milpitas, CA, USA).

### 4.8. Collection of Total Saliva

Rats were anesthetized with an intraperitoneal injection of ketamine at a dose of 1 mL/kg. Salivation was induced with pilocarpine hydrochloride (0.6 mg/kg, IP). Pre-weighed cotton balls were kept in the oral cavity for 10 min to absorb saliva. Cotton balls were immediately weighed on an electronic balance to prevent moisture loss. Total saliva was calculated from the difference of pre- and post-collection cotton ball mass in grams and converted to millilitres. Salivary flow rate was calculated as microliters per minute.

### 4.9. Salivary Protein Concentration Measurement

Immediately after saliva collection, the total protein concentration in saliva was measured using the Bradford method (Bradford Assay, BioRad, Hercules, CA, USA).

### 4.10. Amylase Assay

Amylase activity was measured using a commercial kit (K711-100, Bio Vision Inc., Milpitas, CA, USA) that uses ethylidene-pNP-G7 as a substrate. Absorbance in the α-amylase activity assay was measured at 405 nm at 25° C by using an auto-analyser (Alcyon 300^®^ Plus, Molecular Devices Corporation, Sunnyvale, CA, USA).

### 4.11. Immunoblotting

Western blotting was performed using submandibular gland homogenates. The homogenate was prepared by homogenizing the submandibular gland tissue in radioimmunoprecipitation assay (RIPA) lysis buffer. The composition of lysis buffer was prepared as described in [57]. Supernatant was collected from the homogenates by centrifugation and proteins were separated on 8–10% polyacrylamide gels using sodium dodecyl sulphate polyacrylamide gel electrophoresis (SDS-PAGE). An equal concentration of protein (30 μg) was loaded in each lane of the gels. The proteins were then transferred to polyvinylidene fluoride (PVDF) membrane using a semi-dry transfer device (Bio-Rad, Hercules, CA, USA). Proteins were blocked with 5% skim milk in Tris-buffered saline (0.137 M NaCl, 0.025 M Tris, pH 7.4) for 1 h and incubated overnight at 4 °C with primary antibodies anti-amylase (1:1000, anti-mouse secondary antibody, Santa-Cruz Biotechnology, Dallas TX, USA), anti-AQP5 (1:1000, anti-goat, Santa-Cruz Biotechnology), anti-GRP78 (1:1000, anti-rat, Santa-Cruz Biotechnology), anti-GADD153/CHOP (1:1000, anti-rabbit, Santa-Cruz Biotechnology), anti-ATF6 α (1:1000, anti-rabbit, Santa-Cruz Biotechnology), anti-p-PERK (1:1000, anti-rabbit, Santa-Cruz Biotechnology), anti-PERK (1:1000, anti-rabbit, Santa-Cruz Biotechnology), anti-p-eIF2α (1:1000, anti-rabbit, Cell Signalling, Danvers, MA, USA), anti-eIF2α (1:1000, anti-mouse, Santa-Cruz Biotechnology), anti-p-IRE1α (1:1000, anti-rabbit, Abcam, Cambridge, UK), anti-IRE1α (1:1000, anti-rabbit, Cell Signalling, MA, USA), anti-XBP-1 (1:1000, anti-rabbit, Santa-Cruz Biotechnology), anti-PDI (1:1000, anti-rabbit, Cell Signalling, MA, USA), anti-ERO1α (1:1000, anti-rabbit, Cell Signalling, MA, USA), or anti-β-actin (1:1000, anti-mouse, Santa-Cruz Biotechnology). Total and phosphorylated forms of antibodies were diluted in 5% skim milk and 5% BSA, respectively, prepared in Tris-buffered saline (0.137 M NaCl, 0.025 M Tris, pH 7.4) containing 0.1% Tween-20 (TBST). Secondary antibodies were diluted at a ratio of 1:5000 in 5% skim milk and membranes incubated for 1 h at room temperature. Proteins were visualized using enhanced chemiluminescence (ECL) reagents (Dae Myung Science Co., Ltd. Seoul, Korea) and exposed to imaging film (Kodak BioFlex Econo Scientific Supplies, Citrus Heights, CA, USA). Imaging film was developed by using a Kodak X-OMAT 1000A processor.

### 4.12. Immunoprecipitation

Immunoprecipitation was performed using submandibular gland homogenates as described previously [57]. Antibodies such as amylase (Santa-Cruz Biotechnology), AQP5 (Santa-Cruz Biotechnology) and PDI (Thermo Fisher Scientific, Waltham, MA, USA) were used for the immunoprecipitation experiment.

### 4.13. Haematoxylin and Eosin Stain

Formalin-fixed submandibular gland tissues were routinely processed and embedded in paraffin wax. Tissues were then sliced in 5 μm thick sections. The sections were deparaffinised and rehydrated using standard techniques and stained with Mayer’s haematoxylin and 1% eosin. Stained sections were dehydrated and cleared with xylene. Mounting was done by using anhydrous mounting media (Merck, Darmstadt, Germany) and observed in a light microscope.

### 4.14. Immunohistochemistry

The formalin-fixed, paraffin-embedded submandibular gland tissue sections were sliced at 5 μm thickness, deparaffinised, and subjected to 1× Target Retrieval Solution, pH 6.0 (DAKO, Glostrup, Denmark). Sections were incubated with peroxidase blocking solution (DAKO) for 10 min at room temperature (RT). They were then washed with 1× TBST buffer (Scytek Lab, Logan, UT, USA) followed by a protein block (0.25% casein in PBS, DAKO) for 10 min at room temperature. Primary antibodies including anti-amylase (1:200, anti-mouse secondary antibody, Santa-Cruz Biotechnology), anti-AQP5 (1:100, anti-rabbit, Abcam, Cambridge, UK), anti-NHE1 (1:100, anti-rabbit, Santa-Cruz Biotechnology), anti-GRP78 (1:100, anti-mouse, Santa-Cruz Biotechnology), anti-GADD153/CHOP (1:100, anti-rabbit, Santa-Cruz Biotechnology, USA) or anti-PDI (1:100, anti-mouse, Enzo life sciences, Farmingdale, NY, USA) were diluted in antibody diluent provided by DAKO and incubated in a humidified chamber overnight at 4 °C. Slides containing tissue sections were further rinsed in TBST buffer and incubated with indicated secondary antibodies for 1 h at RT. AEC substrate chromogen (DAKO) was added and washed with deionized water. This was followed by counter staining with Mayer’s haematoxylin (Sigma-Aldrich). The slides were rinsed with tap water and mounted using an aqueous medium (Scytek Lab, USA).

### 4.15. Double Labelled Immunofluorescence

Double-label immunofluorescence was performed using amylase and AQP5 antibody. First, the paraffin-embedded submandibular gland sections were deparaffinised in different changes of xylene and rehydrated with different grades of ethanol. Antigens were retrieved using retrieval solution (DAKO) and treated with peroxidase and a protein block solution (DAKO) for 10 min each at RT. Sections were then incubated with primary antibodies anti-amylase (1:200, Santa Cruz Biotechnology, USA) or anti-AQP5 (1:200, Abcam, Cambridge, MA, USA) overnight at 4 °C. They were then washed in TBST two times and incubated with fluorescein isothiocyanate (FITC)-conjugated anti-mouse secondary antibody (1:300, Sigma) or tetramethylrhodamine isothiocyanate (TRITC)-conjugated anti-rabbit secondary antibody (1:300), respectively, for 1 h at room temperature. This was followed by washing with TBST (5 min, 2 changes each) and mounted with Vectashield mounting medium (Vector Laboratories). Fluorescence was visualized by using FITC and TRITC channels in a confocal microscope (Olympus, Japan).

### 4.16. TBARS Assay

Malondialdehyde (MDA) is an end product of polyunsaturated fatty acid oxidation reacting with TBA to produce a complex that can be spectrophotometrically determined. Lipid peroxidation in samples was assessed by the measurement of produced thiobarbituric acid reactive substances (TBARS). Submandibular gland tissue homogenates were used to measure lipid peroxidation and measured using the OxiSelect™ TBARS Assay Kit (Cell Bio labs, Inc., CA, USA). Briefly, the supernatant obtained from submandibular gland tissues was assayed for TBARS amount. It was analysed using a 96-well microplate, compatible with a spectrophotometric plate reader, with absorbance read at 532 nm.

### 4.17. Salivary Carbonyl Assay

Salivary carbonyls were detected using an OxyBlot Protein Oxidation Detection Kit (S7150, Chemicon). Briefly, after saliva collection, 30 μg of saliva protein were loaded and run on 10% SDS-PAGE. The proteins were then transferred to PVDF membrane using a semi-dry transfer device and blocked with buffer containing 1% BSA in PBS, pH 7.2–7.4 for 1 h. Blots were then incubated with specific anti-dinitrophenylhydrazine (DNPH) antibodies (1:150). Proteins were visualized using enhanced chemiluminescence reagents and exposed to an imaging film.

### 4.18. Statistical Data Analysis

Data are presented as mean ± standard error of the mean (SEM). The statistical software Origin (Origin-Lab Corporation, Northampton, MA, USA) was used for data analysis. Statistical analysis was performed using one-way analysis of variance (ANOVA) and Tukey’s test was done for the individual means. A *p* value of <0.05 was set as the criteria for statistical significance.

## 5. Conclusions

**Figure 8:**
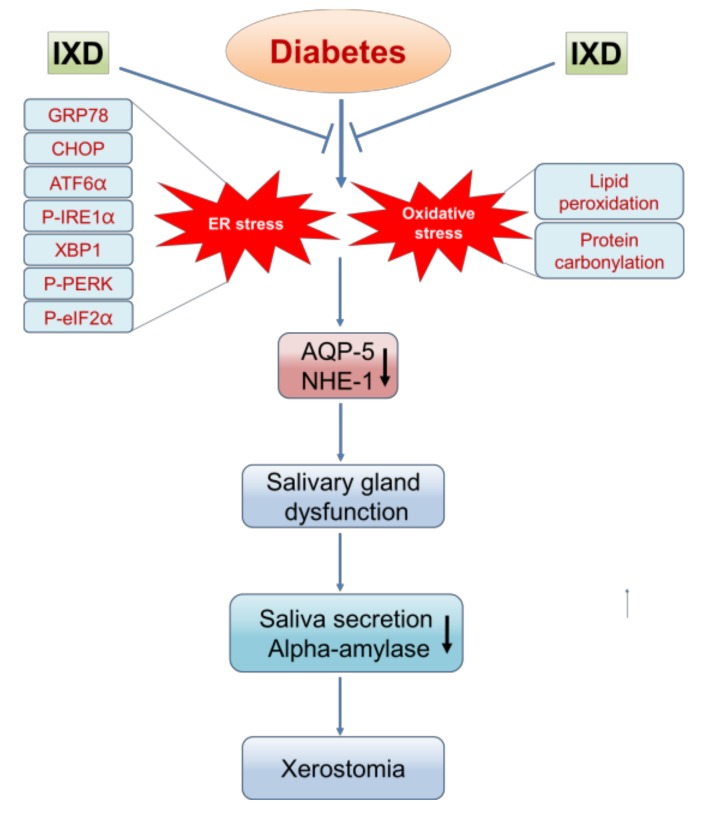
The proposed mechanism of an IXD extract against diabetes-induced xerostomia. The schematic diagram shows diabetes-associated ER stress and oxidative stress, subsequently, altering the protein expression of AQP5 and NHE1 in the submandibular gland. This may cause salivary gland dysfunction with reduction in salivary secretion leading to xerostomia. This graphic shows that IXD extract increases salivary secretion through the regulation of ER stress and prevents from xerostomia.

The important finding of this study with regard to salivary secretion suggests that the IXD extract shows an ability to increase saliva secretion in STZ-induced diabetic rats (Figure 8). Salivary fluid secretion is dependent on the activation of water forming channel proteins. Perturbations in the ER domain increase salivary gland dysfunction leading to hyposecretion of saliva. Dehydration caused by hyposecretion increases xerostomia and polydipsia and worsens oral health, inevitably increasing the risk of oral health complications. The IXD extract has been observed as a potential modulator for salivary secretion. This study has shown, for the first time, a link between ER stress and saliva secretion and reveals a mechanism for the possible treatment of dry mouth with the IXD extract. Genetically modified animal models could be used to further study IXD applications and the treatment of xerostomia.

## Supplementary Materials

Supplementary materials can be found at http://www.mdpi.com/1422-0067/19/4/1059/s1.

## Abbreviations

STZ	Streptozotocin
DM	Diabetes mellitus
IXD	*Ixeris dentata*
AQP5	Aquaporin-5
ER	Endoplasmic reticulum
GCT	Granular convoluted tubule
NHE1	Sodium/Hydrogen exchanger-1
GRP78/BiP	Glucose regulated protein-78 kDA/Binding immunoglobulin protein
GADD153/CHOP	Growth arrest- and DNA damage-inducible gene 153/CCAAT enhancer binding protein homologous protein
p-eIF2α	Phosphorylated eukaryotic translational initial factor 2 alpha
p-PERK	Phosphorylated protein kinase R like endoplasmic reticulum kinase
ATF6α	Activating transcription factor 6 alpha
p-IRE1α	Phosphorylated inositol-requiring enzyme 1 alpha
sXBP1	Spliced X box binding protein 1
JNK	c-Jun N-terminal kinase
PDI	Protein disulphide isomerase
ERO1α	Endoplasmic reticulum oxidoreductin 1 alpha
CBB	Coomassie Brilliant Blue
NaCl	Sodium Chloride
ERAD	Endoplasmic reticulum associated protein degradation
EDEM	Endoplasmic reticulum degradation enhancing alpha mannosidase like protein
SERCA	Sarco/endoplasmic-reticulum Ca^2+^-ATPase
HSP	Heat shock protein
GSH	Glutathione
IP	Intraperitoneal
SDS-PAGE	Sodium dodecyl sulphate polyacrylamide gel electrophoresis
PVDF	Polyvinylidene fluoride
FITC	Fluorescein isothiocyanate
TRITC	Tetramethylrhodamine isothiocyanate
MDA	Malondialdehyde
TBARS	Thiobarbituric acid reactive substances
SEM	Standard error mean
ANOVA	Analysis of variance
