# Supplementary material for: Ixeris dentata Extract Increases Salivary Secretion through the Regulation of Endoplasmic Reticulum Stress in a Diabetes-Induced Xerostomia Rat Model
Source: Int J Mol Sci. 2018 Apr 2;19(4):1059. doi: 10.3390/ijms19041059 (PMC5979381; doi:10.3390/ijms19041059)
Supplement: Supplementary file 1 [file ijms-19-01059-s001.pdf]

International Journal of
Molecular Sciences

Supplementary Materials

A

HPLC conditions			
Instrument Parameter	Agilent 1260 UPLC system		
Column	YMC Hydrosphere C18 (4.6 × 250 mm, 5 μm)		
Mobile Phase	Time	0.1% acetic acid	Acetonitrile
	0.0	93.0	7.0
	13.0	89.0	11.0
	30.0	82.0	18.0
	45.0	72.0	28.0
	55.0	72.0	28.0
Flow rate	1.0 mL/min		
Injection volume	10 μL		
Detection	UV 254 nm		

B

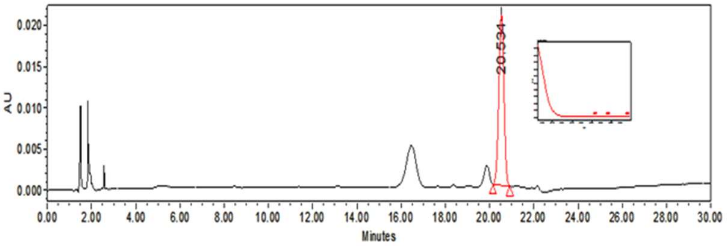

Fig. Ixerin M chromatogram

C

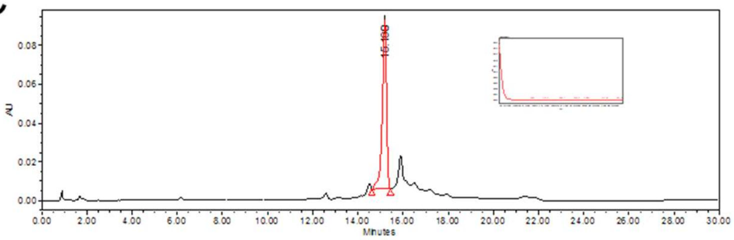

Fig. Ixerin F chromatogram

D

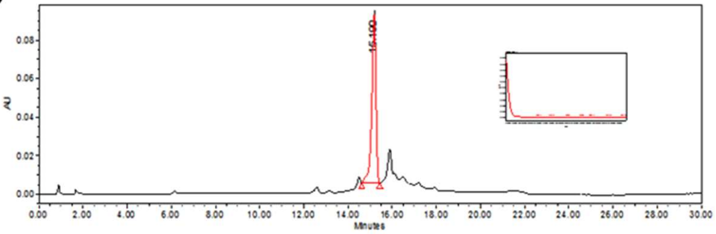

Fig. 8-epiisolipidiol-3-β-D glucopyranoside (8-EI-3-G) chromatogram

Figure S1. HPLC conditions and the chromatogram. (A) HPLC conditions used in quantitation of pure compounds from the IXD extract; (B) Chromatogram of Ixerin M; (C) Chromatogram of Ixerin F; (D) Chromatogram of epiisolipidiol-3-β-D-glucopyranoside.

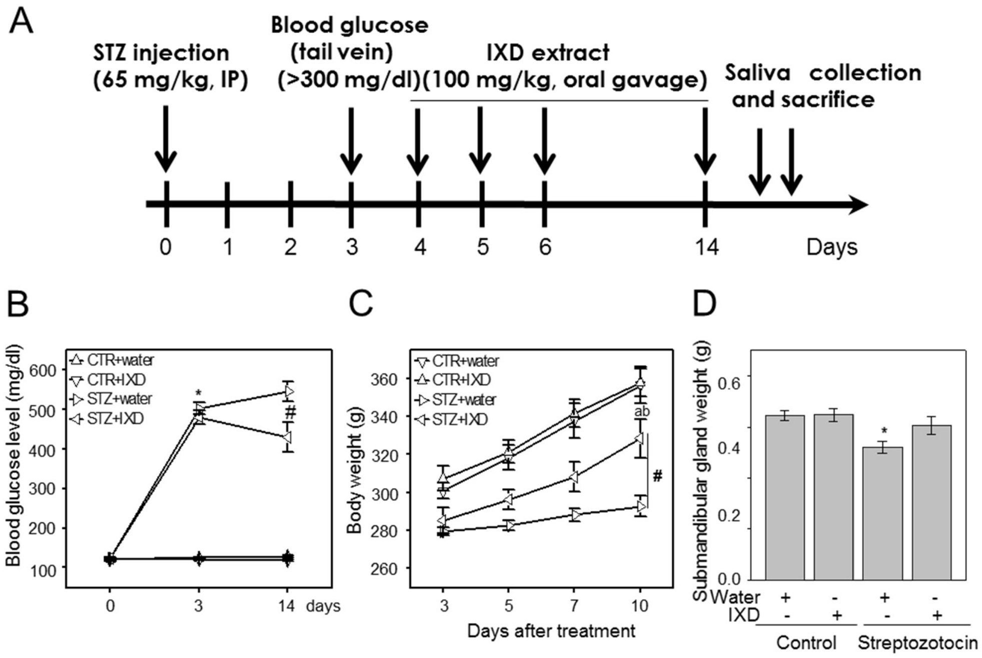

Figure S2. Experiment scheme, blood glucose and body weight measurement. (A) Schematic diagram of the experimental design (*in vivo*); (B) Blood glucose measurement in control and STZ-induced diabetic rats with or without IXD treatment. Diabetes was induced by a single dose of STZ injection. Blood glucose level was determined after 3 days of diabetic induction. Vehicle (water) or the IXD extract was added orally for 10 days, and blood glucose level was measured before saliva collection. * indicates significant differences in STZ-induced diabetic rats compared to vehicle-treated control rats and # indicates significant differences in IXD treated diabetic rats compared to vehicle-treated diabetic rats. Values are represented as mean ± S.E. ($p < 0.05$); (C) Body weight was measured after 3 days of vehicle or STZ injection along with water or IXD extract treatment for 10 days. "a" indicates significant differences in body weight of 10 day- to 3 day-IXD-treated diabetic rats, "b" indicates significant differences in body weight of 10 day- to 5 day-IXD-treated diabetic rats and # indicates the significant differences in body weight of 10 day-IXD-treated diabetic rats to 10 day-vehicle-treated diabetic rats; (D) Total submandibular gland weight (g), *$p < 0.05$.

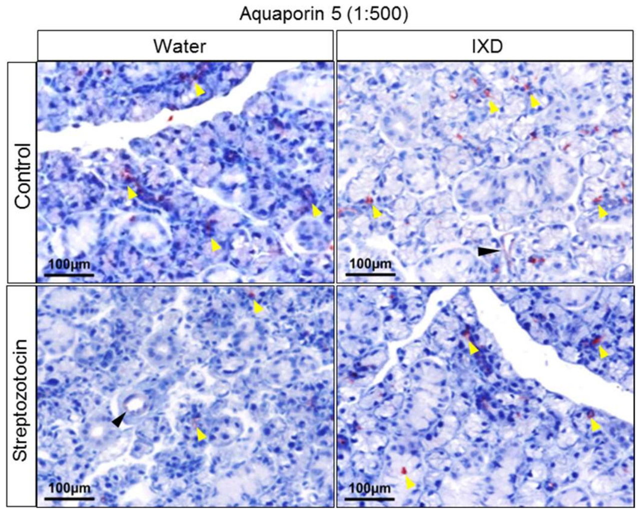

Figure S3. Immunohistochemical detection showing AQP5 protein expression in rat submandibular gland tissue. Immunostaining was performed using anti-rabbit AQP5 antibody at a dilution of 1:500.

Yellow arrow heads pointing to brownish red colour indicate AQP5 positive acinar cells and black arrow heads indicates AQP5 expression in duct cells. Magnification: 40×; Scale bar: 100 µm.

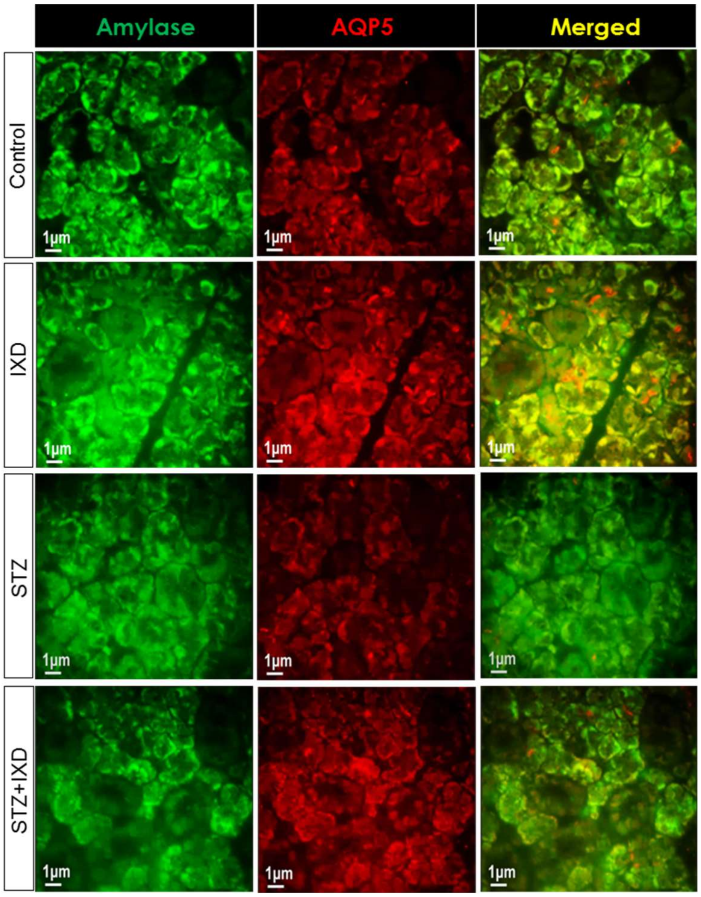

Figure S4. Subcellular localization of α-amylase and AQP5 in the submandibular gland. Double labelled immunofluorescence, performed using amylase and AQP5 antibody, was observed by confocal microscopy. Vehicle or STZ-induced diabetic rats were treated with either water or the IXD extract to observe the expression and localization of amylase and AQP5 in the submandibular gland. Green colour fluorescence indicates α-amylase expression, red colour fluorescence indicates AQP5 expression, and yellow colour fluorescence indicates co-localization of both proteins. IXD extract treatment showed high intensity of amylase and AQP5 fluorescence when compared with their control counterparts. Magnification: 40×; Scale bar: 1 µm.

Table S1. Mass of IXD roots (grams) and extracts obtained, calculated in grams and percentage.

	20% EtOH	40% EtOH	60% EtOH	80% EtOH	100% EtOH
Total IXD root weight (g)	1150	1200	1200	1200	1200
IXD extract (g)	36.23	27.44	29.75	22.92	30.54
Extract (%)	3.15	2.29	2.48	1.91	2.55
